# Measuring Medicaid Expansion’s Impact on an Academic Medical Center’s Emergency Department

**DOI:** 10.7759/cureus.3798

**Published:** 2018-12-31

**Authors:** Vikrant Bhatnagar, Sebastian R Diaz, Susan D Moffatt-Bruce

**Affiliations:** 1 Family Medicine, Ohio University Heritage College of Osteopathic Medicine, Athens, USA; 2 Surgery, The Ohio State University Medical Center, Columbus, USA

**Keywords:** academic medical center, emergency medicine, health policy, medicaid expansion, performance evaluation/improvement

## Abstract

Medicaid expansion’s impact has been studied on national and statewide levels with respect to the patient outcomes, access to health services and uncompensated care. The objective of this study was to examine the effect of the Medicaid expansion on an emergency department (ED) at a large, academic center by evaluating changes in total charges, services rendered, types of providers, number of visits, and length of stay. Findings from this study include more males frequenting the ED for health services and a bottleneck in operations with an average waiting time in the ED increasing by 17%. Additionally, Medicaid recipients required non-emergent services that could be delivered by primary care providers, albeit at the ED, with average total charges for Emergency Medicine services seeing a statistically significant reduction with increases in average total charges for Family Medicine. While Medicaid expansion provided more individuals with coverage, a large academic medical center adapted in concordance.

## Introduction

One of the tenets of the Patient Protection and Affordable Care Act [1] was to expand state-run Medicaid programs via the allocation of federal funds. Non-elderly adults below 138% of the federal poverty line would become eligible under this law [2]. On January 1st, 2014, 27 states, including Ohio, adopted this expansion. During the first year, newly eligible enrollees (n = 448,378) gained coverage in Ohio. By the end of 2016, this number rose to 633,949 [3].

Research on increasing access to health insurance has addressed the scope and depth. A 6.1% reduction in adjusted all-cause mortality was found in states that expanded Medicaid coverages relative to non-expansion states [4]. States that expanded Medicaid coverage saw a reduction in the number of uninsured individuals, improvements in self-reporting of health, and increased access to care previously delayed by cost [5-9]. Expansion of coverage was found to increase the rate of diagnosis and treatment of chronic conditions [7-8, 10].

While a subset of the population gained insurance coverage, the benefits of this expansion were also experienced by hospitals. Analysis of public and private hospitals in Connecticut demonstrated a 7% - 9% increase in Medicaid discharges, a 7% - 8% boost in Medicaid’s share of revenue, and a 33% decrease in uncompensated care [11]. Estimates suggest that a 30% reduction in uncompensated care would lead to a 1.7% decrease in operating costs (5.7% to 4.0%) [12], benefiting hospitals two-fold by reducing the amount of bad debt and increasing operational efficiency.

One objective of this government-funded expansion of insurance coverage was to encourage utilization of health services in a non-emergent setting, preferably at a primary care facility, rather than visiting the emergency department (ED) for these healthcare needs [13-14]. The current literature is inconclusive on how increased coverage like Medicaid expansion impacts ED visits. Data from the Agency for Healthcare Research and Quality’s Fast Stats found that in 2014 expansion states experienced an increase of 2.5 ED visits relative to non-expansion states [15]. Another analysis found a 29% relative decrease in ED visits in expansion states compared to non-expansion states [16].

While these analyses utilized data from statewide, publicly available databases, there is sparse literature that empirically evaluates the effect of the Medicaid expansion on ED visits using data held by individual hospitals. Access to proprietary data can shed light on the delivery of care with an influx of patients. The objective of this study was to understand the changes in (1) total charges, (2) services rendered, (3) types of providers, (4) number of visits, and (5) length of stay from prior to and after Medicaid expansion in Medicaid recipients visiting the ED at a large academic medical center.

## Materials and methods

From January 1, 2011, to December 31, 2016, the ED at a large Midwestern academic medical center (AMC) saw patients from all financial classes (n = 645,765), out of which 257,252 (39.8%) were inpatients with Medicaid coverage. Using business intelligence tools and electronic medical record administrative tables and data sources, 31,310 (12.2%) unique Medicaid billings were randomly extracted. This extraction yielded 98,244 total patient encounter billings. Patient billings were then split by year: 2011 (n = 4,669), 2012 (n = 17,840), 2013 (n = 22,180), 2014 (n = 25,472), 2015 (n = 22,073), and 2016 (n = 6,010). The years 2011 to 2013 were categorized as the pre-expansion group (n = 44,689), while 2014 to 2016 were aggregated as the post-expansion group (n = 53,555).

Data on patient visits included categories, such as patient identifier, unique encounter code, age, gender, race, arrival time, admit time, discharge time, length of stay, primary diagnosis, problem list, specialty utilized, total charges, ED readmission, and in-patient readmission. With admission encounters as a base, readmissions were counted as the number of times a patient was readmitted to the ED or the hospital within three days of a previous discharge. All patient lists were programmatically de-identified to preserve patient privacy and this de-identified dataset was achieved through the honest broker system. Due to the nature of this research project (healthcare operations assessment), institutional review board (IRB) approval was not required; however, IRB exemption was received through the Data Quality Request process.

Using de-identified abstraction identifiers, 6,675 (21.3%) patients were found to have utilized Medicaid in both the pre- and post-expansion eras and, therefore, were not considered as new enrollees from the Medicaid expansion. Aggregation of these patients was used to approximate readmission rates.

To appreciate the changes in the cost of services rendered by the ED, total charges were normalized to account for the compounding effect of inflation. Using the consumer inflation rates from the Bureau of Labor Statistics from 2011-2016, the total charges subsequent from 2011 were normalized to the value of the dollar in 2017. Year-to-year inflation rates from 2011 to 2016 were noted to be 1.7%, 2.1%, 1.8%, 1.8%, 1.8%, and 2.2%, respectively [17].

Inferential statistical analysis of the database was conducted using the Statistical Package for Social Sciences (SPSS) Statistics, version 25.0 (IBM SPSS Statistics, Armonk, NY). The analysis of variance (ANOVA) was employed to determine the statistical significance between the means of two or more groups. The Scheffé test was used to explore post hoc multiple comparisons for ordinal independent variables. Statistical significance was determined by using the p = 0.05 cutoff.

## Results

**Figure 1:**
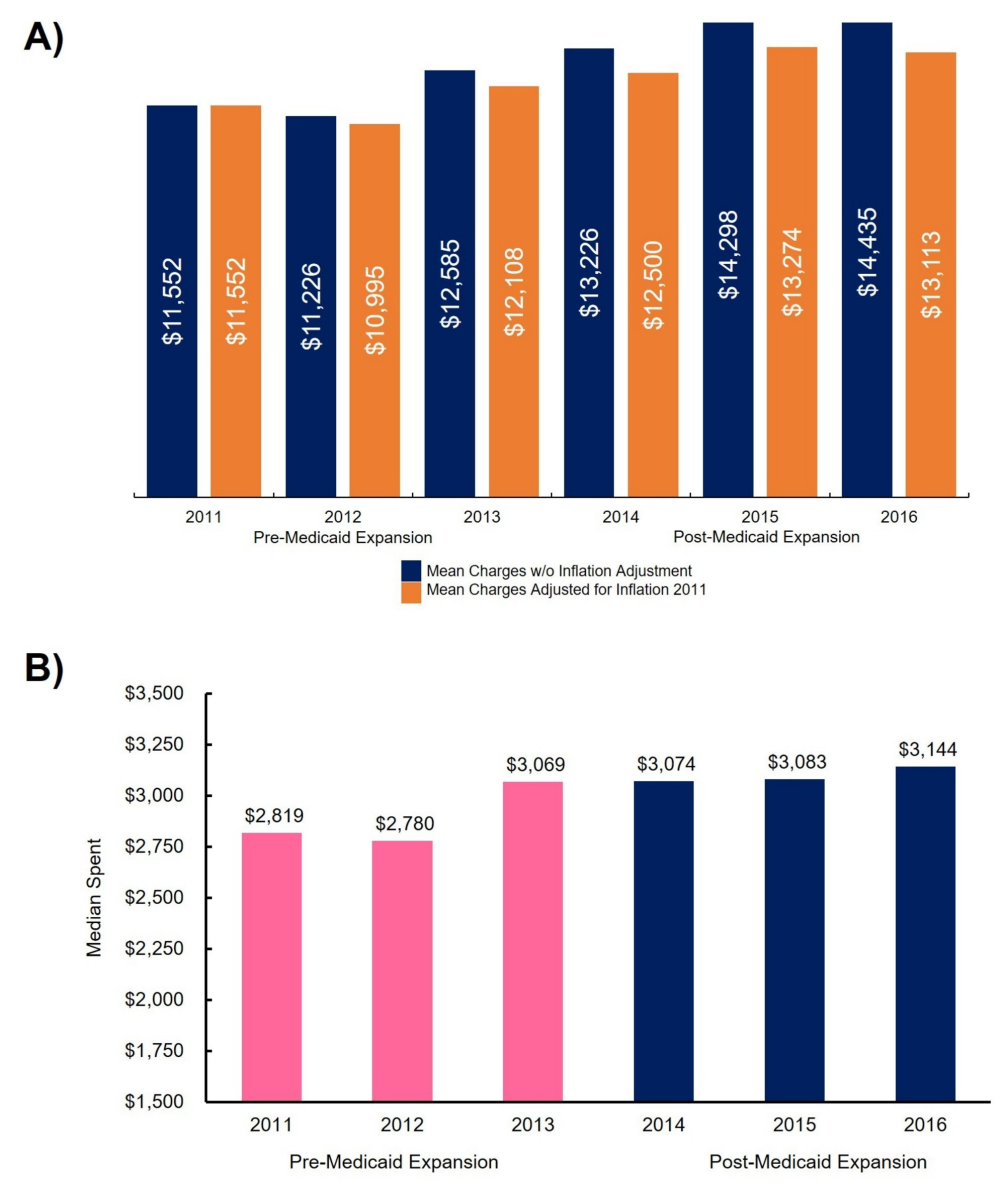
Cost of services A) Mean cost of services rendered to Medicaid recipients from 2011 to 2016, with reported charges (blue) and charges adjusted for inflation (orange); B) median cost of services provided to Medicaid recipients from 2011 to 2016, with pre-Medicaid expansion (2011 - 2013) in pink and post-Medicaid expansion (2014 - 2016) in blue.

This study found that the mean cost of care for Medicaid recipients steadily increased from 2011 to 2016, with a slight decrease in 2012. Figure 1A illustrates the mean total charges with and without the inflation adjustment. The Scheffé test found statistical differences between the mean costs of 2012 and 2014 (p = 0.005), 2012 and 2015 (p < 0.001), 2012 and 2016 (p = 0.015). Median total charges exhibited a similar increasing trend, except in 2012, and a plateau in median costs was detected from 2013 to 2015 (Figure 1B).

**Figure 2:**
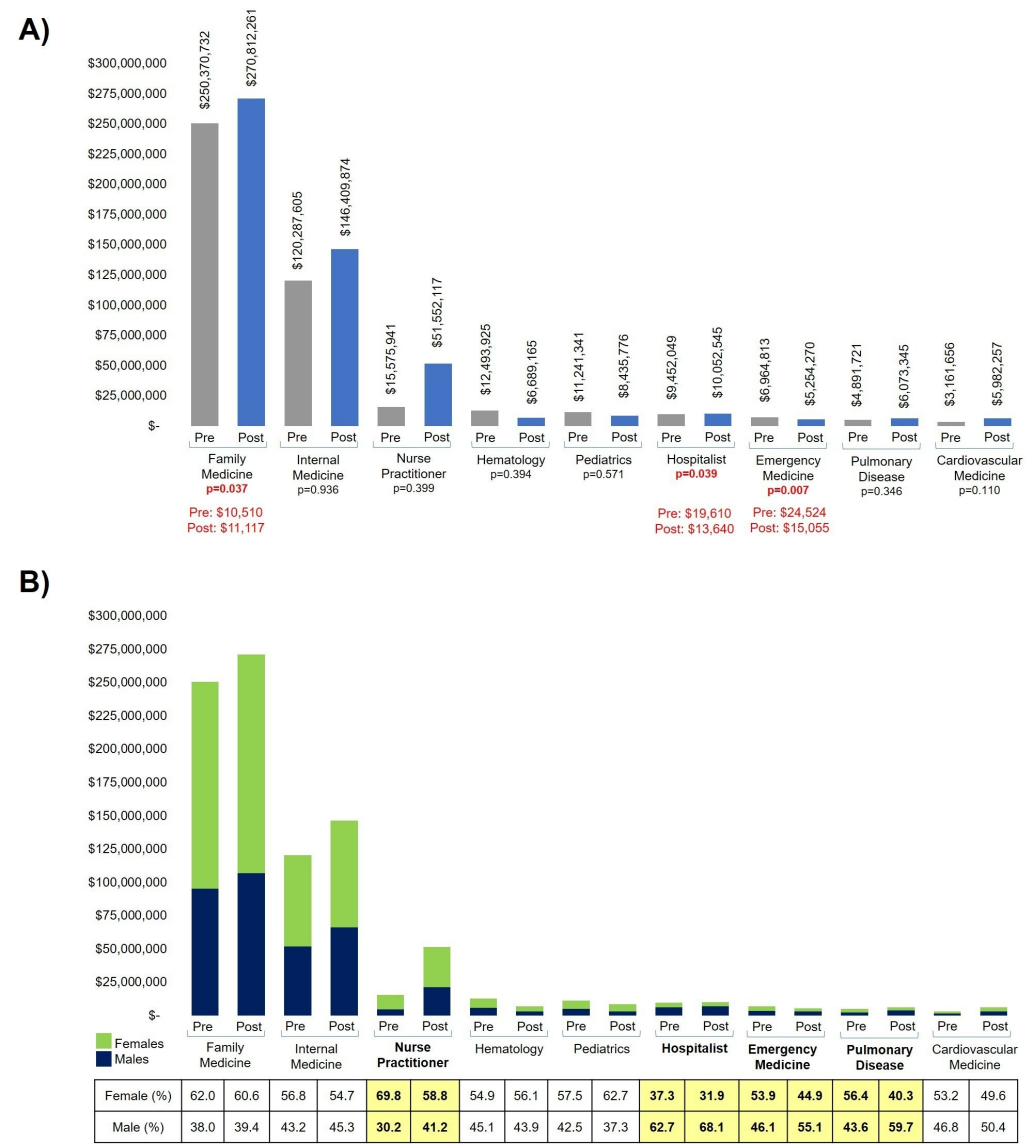
Care rendered by specialties Only specialties with greater than $5 million total charges for either pre- or post-expansion group are shown. A) Total charges accrued by each type of specialty are plotted by pre- (gray) and post-expansion (blue) areas. Family Medicine, Hospitalists, and Emergency Medicine were the only specialties found to have statistically significant differences (seen in red); B) separation of total charges by gender for each specialty, females in green and males in blue. Nurse Practitioners, Hospitalists, Emergency Medicine, and Pulmonary Disease were specialties that had a greater than 5% change in utilization by males.

Aggregating total charges by specialties and comparing pre- and post-expansion groups provided insight as to the utilization of ED services and the types of providers needed to render these services. Total charges increased in Family Medicine, Internal Medicine, Nurse Practitioners, Hospitalists, Pulmonary Disease, and Cardiovascular Disease, with a reduction in charges seen in Hematology, Pediatrics, and Emergency Medicine. Statistically significant changes between the average total charges from pre and post-expansion groups were found in Family Medicine ($10,510 to $11,117; p = 0.037), Hospitalists ($19,610 to $13,640; p = 0.039), and Emergency Medicine ($24,524 to $15,055; p = 0.007). Specialty categories with an aggregation greater than $5 million were plotted in Figure 2A.

Family Medicine, Internal Medicine, and Nurse Practitioners were the three largest specialties by total charges combined. Separating each specialty by gender utilization found that prior to Medicaid expansion, females’ utilization of each specialty, except Hospitalists, was greater than 50%. However, post-expansion, male utilization greater than 5% was seen in Nurse Practitioners, Hospitalists, Emergency Medicine, and Pulmonary Disease (Figure 2B). Relative changes from pre- to post-expansion eras highlight a substantial increase in the usage of Nurse Practitioners for both genders. There was decreased utilization of Emergency Medicine, Pediatrics, and Hematology services in both genders.   

**Figure 3:**
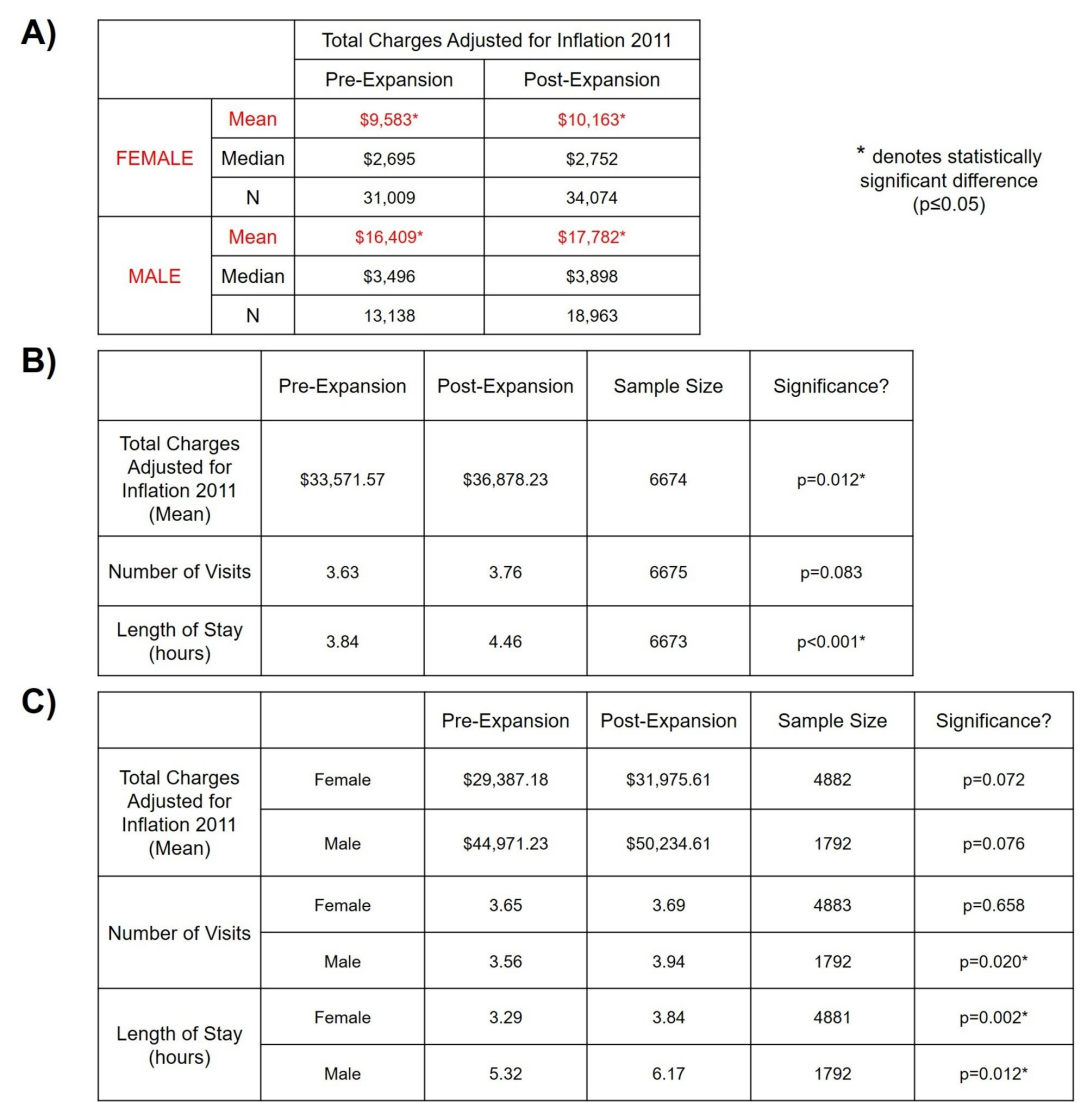
Gender differences A) Separating total charges by gender illustrates statistically significant differences in the mean charges from pre- to post-expansion eras; B) individuals who utilized Medicaid services in both pre- and post-expansion eras were utilized as a proxy to appreciate changes in total charges, visitation frequency, and length of stay (in hours); C) individuals from 3B were separated by gender to appreciate changes in total charges, visitation frequency, and length of stay (in hours).

Separating total charges by gender found that the average cost accrued by females increased from approximately $9,583 to $10,163 from pre- to post-expansion era, with males increasing from $16,409 to $17,782 (Figure 3A). This difference in means was noted to be statistically significant (p < 0.001). Therefore, males were found to be 1.71 - 1.75 times costlier than females in the pre- and post-expansion eras. Males had a greater percentage change (8.4% to 6.1%) in the mean total charges from pre- to the post-expansion era and exhibited higher median total charges in both eras. While females had a higher number of patient visits, the relative percentage of visits by females decreased from 70.2% to 64.3% from pre- to post-expansion era as males experienced a 44.4% increase in the same period.

Using unique patient identification, 6,675 individuals utilized Medicaid services in both pre- and post-expansion groups. This group served as a proxy to appreciate changes in the total charges, visitation frequency, and length of stay (in hours). The post-expansion group saw increases in the number of visits, total charges, and length of stay with statistically significant differences found in the latter two (Figure 3B). Analyzing these variables by gender illuminated that while total charges did increase for both genders, they were statistically non-significant (p = 0.072 for females; p = 0.076 for males). The number of visits increased for both genders; however, statistically significant differences were only seen in males (p = 0.020). Statistically significant increases in length of stay were seen in both genders (p = 0.002 for females; p = 0.012 for males). Figure 3C illustrates these differences by gender.

**Figure 4:**
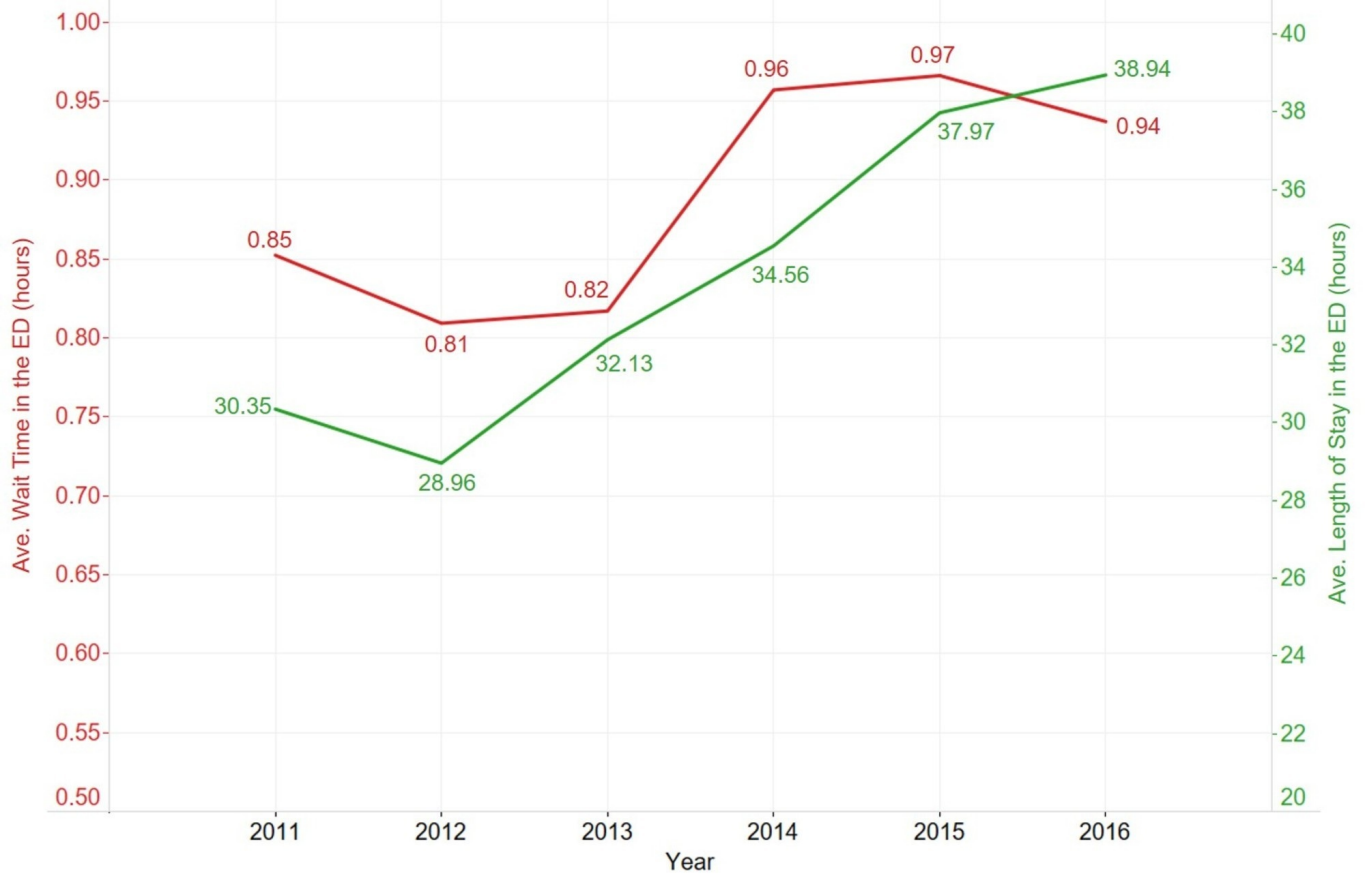
Waiting times Trends in average waiting time (red) and average length of stay in the ED (green) are illustrated. ED: emergency department

Healthcare delivery was measured by the average waiting time in the ED, which increased by 17% (0.82 hours to 0.96 hours) or 10.24 minutes from 2013 to 2014 at the approximate time Medicaid expansion went into effect (Figure 4). The average length of stay in the ED measured in hours steadily increased from 2012.

**Figure 5:**
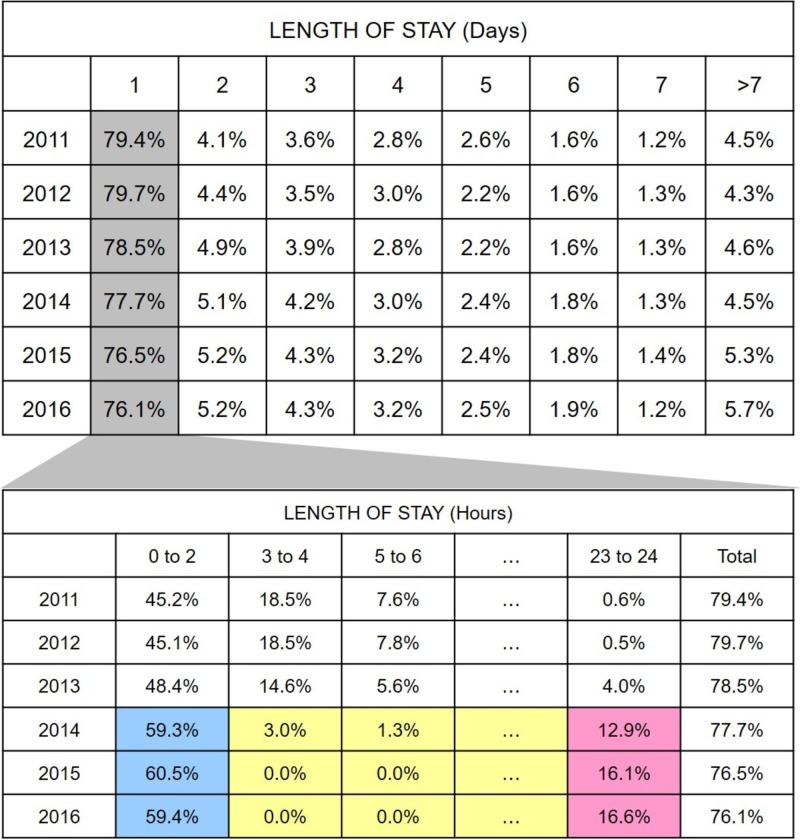
Length of stay Percentage of total visits categorized by their length of stay (days). Stays longer than seven days were grouped into one category. Splitting Day 1 into two-hour periods showcases the changes in discharge patterns, with the post-expansion era seeing an increase in length of stay in the first (blue) and last (pink) two hours and decreases in hours 3 to 22 (yellow).

When the length of stay is measured in days, post-expansion years saw a decrease in the length of stay on Day 1 but increases in Days 2-4 were seen. Splitting Day 1 (gray in Figure 5) into two-hour slots provided additional insights: 1) post-expansion years saw a substantial increase in the length of stay in the first two hours (blue in Figure 5); 2) length of stay was much lower in the post-expansion years from hours 3 to 22 (yellow in Figure 5); and 3) length of stay in the last two hours of the day was much higher in post-expansion years (pink in Figure 5).

Limitations

There are several limitations to the study. First, this was a single site study that examined the impact of Medicaid expansion on ED visits. While some of the findings from this study may be site-specific, there are findings consistent with the current literature, such as more males seeking healthcare services at the ED upon receiving Medicaid coverage [18] and differences in healthcare utilization between the genders [19-20]. External validity potentially suggests that this limitation would not substantially change the results of our study. Additionally, the findings from this study add to the current literature, which, to the best of our knowledge, currently does not include an analysis of the impact of Medicaid expansion on a specific hospital.

A second limitation was only 12.2% (31,310 out of 257,252) of the total possible Medicaid billings from 2011 to 2016 were studied. It should be noted that the inclusion of the other 87.8% of billings could potentially affect some of our findings and unveil new findings, currently undetected or underappreciated. However, random extraction of billing data can help reduce biases in the evaluation of data while providing an adequate sample size to parse out differences between genders and services utilized from the pre and post-Medicaid expansion groups in a statistically significant manner.

The third limitation of this study includes the impact of variables that were outside the control of this study. For example, demographic changes in the patient population could possibly affect the utilization of Medicaid services. These changes could potentially impact the perception of Medicaid, willingness to seek out care, and follow-up with a provider [18]. Furthermore, additions/subtraction of emergency services in other surrounding hospitals, regionally or within the state, were outside the control of this study. These changes could affect the findings of this study as they would impact patient demographics and healthcare delivery in the form of time spent waiting in the ED and the total length of stay.

## Discussion

Using hospital-specific data for Medicaid patient encounters between 2011 to 2016, while analyzing trends for a pre- and post-expansion state, provided actionable insights on Medicaid expansion’s impact on an ED at a single, large academic medical center. No statistically significant changes were found in the mean total charges from 2013 to 2015 (representing the post-expansion time period). A plateau in the median costs from 2013 to 2015 was detected (Figure 1B), suggesting the initial effect of Medicaid expansion included stemming the costs of the medical services rendered. This result is intriguing when considering the “pent-up” demand, albeit temporary, for medical services in a previously uninsured segment of the population that did not statistically change the median cost [18, 21].

Findings from this case analysis suggest that coverage via Medicaid expansion potentially decreased a barrier to care for males, with more males seeking healthcare services at the ED post-expansion. This finding is consistent with the analysis of the National Health Interview Survey (NHIS) which found expansion states to cover 1.8% more males than non-expansion states (p = 0.01) [18]. Considering that males were found to be 1.71 - 1.75 times costlier than females, this increased cost can be potentially attributed to the severity of disease elongating the length of stay in the hospital and differences in the perception of healthcare utilization between males and females [19-20].

Findings from this study indicate that, upon expansion, more individuals frequented the ED, less for the actual ED service but rather more for primary care services. The existence of this pattern prior to Medicaid expansion [13] allowed the academic center in this study to potentially anticipate increases in the types of services and staffing the ED with providers accordingly. Emphasis was placed on adding providers in fields like Family Medicine, Internal Medicine, and General/Specialized Nurse Practitioners to provide primary care services. This subsequently increased the total charges for Family Medicine practitioners, while decreasing the total charges for Emergency Medicine providers.

It should be noted that increased ED utilization is associated with a higher number of barriers to primary care services, such as a lack of transportation, the timing of the provider’s office, length of time spent waiting at provider’s office, and the next available appointment [22]. Additionally, ED usage has been found to be higher in individuals gaining new coverage compared to individuals with continuous coverage [13], potentially indicating why there was an increase in ED visits. Providers within the ED can be key in coordinating the delivery of care across various practitioners, especially primary care [13].

Medicaid expansion also created bottlenecks in operational management. While proper types of providers were hired, operational processes did not seamlessly handle the influx of patients as an increased average waiting time in the ED was seen (Figure 4). This finding is in concordance with the National Hospital Ambulatory Medical Care Survey, which found a 1.2% increase (66.7% - 67.9%) from 2013 to 2014 in patients waiting between 0 - 59 minutes at the ED [23-24]. Interestingly, a higher percentage of patients were discharged in the first two hours post-expansion. Streamlined operational processes, including an increased number of providers, improved the percentage of patients discharged in the first two hours. This finding is in contrast to the data collected by the National Hospital Ambulatory Medical Care Survey which found a 2.1% decrease (37.3% - 35.2%) of all patients spending between zero to two hours at the ED from 2013 - 2014 [23-24]. More research is needed to better understand if these national trends of increased wait times and decreased discharge percentages were also found in other large academic centers and if these trends hold true to the Medicaid population. Insights gained from such research could further increase operational efficiencies, potentially leading to better delivery of care.

## Conclusions

As Medicaid expansion went into effect and provided more individuals with coverage, a large academic medical center adapted accordingly. This study found five important and actionable insights. First, insurance coverage encouraged more males to frequent the ED for health services. Second, the influx of newly eligible Medicaid patients caused a bottleneck in ED operations with average waiting time increasing by 17%. Third, many of the newly eligible Medicaid recipients required non-emergent services that could be delivered by primary care providers, albeit at the ED. The average total charges for Emergency Medicine saw a statistically significant reduction balanced with an increase in average total charges in Family Medicine. Fourth, the addition of more advanced practice providers, such as nurse practitioners, potentially stemmed the costs of services provided. Fifth and last, while the mean cost of care for Medicaid recipients did not exhibit a clear trend, a plateau in median costs from 2013 to 2015 potentially hints at the impact of Medicaid expansion on curbing the rising costs of healthcare services rendered to Medicaid patients.
